# Paper-Based Screen-Printed Electrodes: A New Generation of Low-Cost Electroanalytical Platforms †

**DOI:** 10.3390/bios11020051

**Published:** 2021-02-16

**Authors:** Estefanía Costa-Rama, María Teresa Fernández-Abedul

**Affiliations:** Department of Physical and Analytical Chemistry, University of Oviedo, Av. Julián Clavería 8, 33006 Oviedo, Spain; costaestefania@uniovi.es

**Keywords:** microfluidics, electroanalysis, paper-based devices (µPADs), paper-based electroanalytical devices (ePADs), screen-printed electrodes

## Abstract

Screen-printed technology has helped considerably to the development of portable electrochemical sensors since it provides miniaturized but robust and user-friendly electrodes. Moreover, this technology allows to obtain very versatile transducers, not only regarding their design, but also their ease of modification. Therefore, in the last decades, the use of screen-printed electrodes (SPEs) has exponentially increased, with ceramic as the main substrate. However, with the growing interest in the use of cheap and widely available materials as the basis of analytical devices, paper or other low-cost flat materials have become common substrates for SPEs. Thus, in this revision, a comprehensive overview on paper-based SPEs used for analytical proposes is provided. A great variety of designs is reported, together with several examples to illustrate the main applications.

## 1. Introduction

In the last decades, the great advances in (micro)electronics, (nano)technology, and material science have led to an easy availability and management of increasing information. In turn, society requires real-time information to deliver an immediate feedback if necessary. In this knowledgeable society, it is obvious the interest in developing low-cost, miniaturized and easy-to-use analytical devices that provide on-site quantitative information in a fast and easy way. The high potential of these devices makes them useful in very assorted fields: from clinical and biomedical applications [1,2,3,4,5] to food analysis and quality control [6,7,8], as well as environmental monitoring [9,10,11,12].

**Figure 1:**
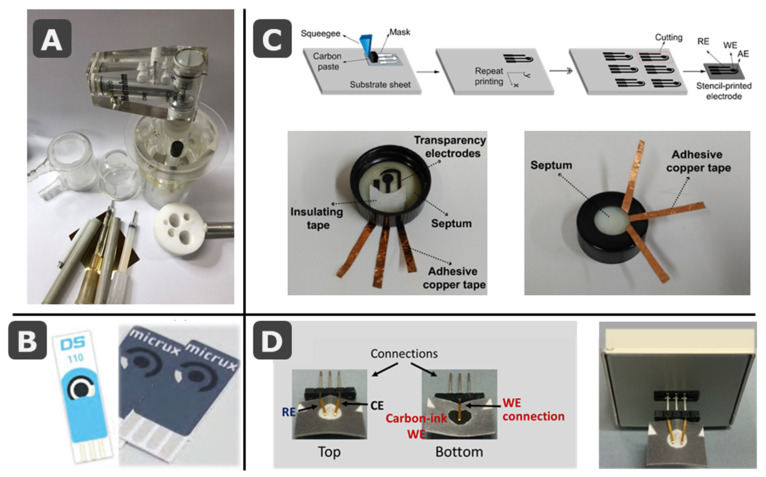
(**A**) Picture of conventional electrochemical cells with pen-like electrodes. (**B**) Examples of commercial screen-printed electrode cards from DropSens (ceramic substrate [13]) and MicruX (polymeric substrate [14]). (**C**) Schematic drawing of the stencil-printing process to fabricate several low-cost electrodes on a transparency sheet, with pictures showing the integration of the transparency electrode onto the cap of a sample vial for on-site water analysis. (**D**) Pictures of a paper-based electrochemical cell containing the three electrodes (top and bottom views), also inserted in a commercial interface that connects electrodes to the potentiostat. (**C**) and (**D**) are reprinted from [15] (Chapters 4 and 25), Copyright (2020), with permission from Elsevier.

As it is well-known, screen-printed electrodes (SPEs) have contributed enormously to the great development of electroanalytical devices. Conventional cells employed in amperometric/voltammetric measurements usually consisted of pen-like electrodes; namely, working (WE), reference (RE), and counter (CE) electrodes in a potentiostatic system of three electrodes. If only two electrodes were employed, apart from the WE, an auxiliary electrode acts as both, RE to apply a stable potential and CE to close the electrical circuit. Before the spread of solid electrodes, a mercury electrode delivered renewable drops from a glass capillary, following also a pen-like format. All the electrodes were introduced in a glass container of ca. 10 or 20 mL of volume (Figure 1A).

This configuration, that resulted appropriate for centralized labs, was associated to an instrumentation (potentiostat) that was also a bench equipment, with clips and cables that usually connected to the top of each pen-like electrode. A stand with mechanized holes was used for adding the solutions and also for nitrogen purging in case deoxygenation was required. Different cell designs were available, even thermostatized. Mass transport was easily controlled by locating the cell over a magnetic stirrer and introducing a stir bar in the cell. Alternatively, rotating rods and electrodes were also applicable. In this way, measurements could be done under forced convection or pure diffusion regimes. Apart from this, flow cells were also accessible, with the WE located in a flow cell with wall-jet or thin-layer configurations as the most common, with RE/CE placed downstream, in a specific container leading to the waste.

The use of thick-film technology (producing layers of thickness in the micrometer scale) employed by electronic engineers (as e.g., screen-printing) using conductive inks allowed the development of flat platforms that included all the three electrodes of the electrochemical cell in the same plane (Figure 1B). This takes advantage of the interfacial nature of electrochemical measurements. Since only a surface able to transfer electrons in one way or another (oxidation or reduction) is required, the thickness is not an important variable. This simplifies enormously the design of the electrochemical cell and expands the possibilities. Then, the 3D-electrode/cell ensemble is converted into a 2D electrochemical cell, where the electrolyte can be deposited in such a way that drop analysis becomes possible. Although these flat cells could also be introduced in a conventional glass container, measurement-on-drop procedures have led the field. This change supposed a very important contribution to Green (Analytical) Chemistry (GAC) [16]. Regarding e.g., pollution detection, this was historically conducted with field sampling protocols that require extensive effort to be brought to a laboratory where extensive work-up generated large volumes of solvents and waste. Therefore, analysis of an environmental problem often contributed to other environmental problems. With the use of real-time in-field analysis, the necessary measurements can be taken without wasting time, material and energy. Actually, most of the 12 principles of GAC [17] are followed by miniaturized electroanalytical devices.

Then, screen-printing technology allows to easily obtain small-size flat electrochemical cells which are robust, cheap, and mass-produced. Alternatively, in many cases, a stencil is used instead of a screen, especially in handmade devices. Therefore, the term stencil-printed electrodes, closely related, is also found in the bibliography. The disposability of platforms avoids polishing/cleaning/activating treatments required by other solid electrodes [18] such as carbon paste [19], glassy carbon [20,21], or noble metal [22] electrodes. Moreover, (bio)assays could be performed in parallel. Although the most common commercial screen-printed electrode (SPE) card contains the three electrodes (WE, RE and CE) of the electrochemical cell, different configurations are possible due to the high versatility in design, one of the most important advantages of SPEs [2,6]. Currently, platforms with more than one electrochemical cell and cells with more than one WE can be found in the market [13,14,23]. Multianalyte determination (with e.g., spatial separation), as well as recording simultaneous measurements of both redox or non-redox active analytes, becomes available, and constitutes a field of enormous interest [24].

There is a great variety of inks that can be used for printing the electrochemical cells. Carbon continues to be the most common because of its good characteristics for electroanalytical applications, although metallic inks based on e.g., gold or platinum are also used [13,25,26]. (Bio/nano)modifiers can also be added, before or after the screen-printing process [2,27,28]. For the RE, silver-based inks are common, resulting in pseudo-reference (literally “false” reference) or quasi-reference (“almost” or “essentially” reference) electrodes [29]. The main difference between true reference and pseudo-reference electrodes is the lack of thermodynamic equilibrium in the latter case, since there is no common component in the two adjacent phases. Apart from their simplicity, and because they are immersed directly into the electrolyte used in the cell, the ohmic drop is small, no liquid junction appears and, usually, there is no contamination of the test solution by molecules/ions that a conventional reference electrode might transfer. Although there are also some drawbacks (e.g., they are not ideally nonpolarizable and work over a limited range of conditions such as pH or temperature), under selected conditions, the potential (although unknown) might be surprisingly constant during experiments. Direct exposure to the test environment could limit its applicability in complex sample matrices, but their short conditioning time allows performing measurements with insignificant potential drift. Moreover, since paper-based devices are often conceived for single-use measurements, the required operational stability of the RE is limited to one unique measurement and not to a large number, as in the case of conventional REs. However, where uninterrupted gathering of sensor data over longer periods of time is required, as in the case of inaccessible or remote locations where sensor replacement is difficult, potential stability has to be thoroughly studied [30].

The increasing use of multimodal detection systems that merge electrochemical and optical techniques such as spectroelectrochemistry, has led to the commercialization of electrochemical cells that combine screen-printed RE and CE with optically transparent WEs made of e.g., indium tin oxide, carbon (made of carbon nanomaterials) or gold (obtained by sputtering) [13]. Regarding the substrate, the electrochemical cell was usually printed on rigid materials such as ceramic [13]; however, nowadays the use of SPEs on flexible polymers are increasingly common, either commercial [14] or homemade [31] (Figure 1B,C, respectively).

Taking into account all the advantageous characteristics of the SPEs (mainly their disposability combined with precision), their success as transducers in electroanalytical devices is understandable. While the use of SPEs on ceramic and polymeric materials became widespread, in 2007, paper was demonstrated as innovative substrate for developing promising microfluidic analytical devices (µPADs) [32]. When compared with conventional microfluidic analytical devices, commonly based on glass or polymers, µPADs result simpler, cheaper, and then disposable. Moreover, paper is a lightweight and flexible material with the ability of transporting liquids without the need of external forces [33,34]. The first µPADs were colorimetric but this detection principle provided just qualitative or semiquantitative information and poor sensitivity [35,36]. However, due to the above mentioned advantages of screen-printing technology for the fabrication of electrodes, it is not surprising that the first microfluidic paper-based electroanalytical device (ePAD) was based on SPEs [37] (Figure 5A). After this pioneering design, electrochemical detection was continuously and easily integrated with paper-based devices because of its ability for miniaturization, low-cost and simplicity of the required instrumentation [38]. In the last years, the development of paper-based electroanalytical devices has experienced an enormous increase and, although other kind of electrodes (e.g., metallic film and wires [39,40,41]) have been also integrated in these devices, paper-based SPEs continue to be among the most reported. SPEs printed on or integrated with paper-based devices have been developed for the construction of different types of biosensors (e.g., enzymatic, immunosensors, DNA/aptasensors), with many different designs and for a wide variety of applications (clinical, food or environmental analysis) [5,11,12,34,35,36,38]. Alternatively, paper and ink can be combined by simple deposition of a conductive carbon dispersion (WE), very useful when both paper faces are employed in the design of the electrochemical cell (Figure 1D) [15,42].

Considering that the field of paper-based analytical devices is currently a very active research area and that SPEs are the most common transducers in biosensors and point-of-need devices, the purpose of this work is to review their integration to produce interesting miniaturized devices with different innovative designs and promising applications. The main approaches and trends in the development of these paper/SPE-based devices will be here discussed.

## 2. Paper as Substrate in (Electro)analytical Platforms

### 2.1. Paper as Material: Some Properties

A rapid development of calligraphy by archaic Chinese scholars, their spontaneous adoption of a camel-hair brush and fluid pigment, together with the urgent need of a writing substance cheaper and more practical than those already used, e.g., woven textile, inspired Ts’ai Lun in the year 105 to invent true paper [43]. It was defined as a thin, felted material formed on flat, porous molds from macerated vegetable fiber. This process separated each individual filament as a unit, and after adding water, fibers were filtered and dried leaving a sheet upon. Paper sheets were very advantageous compared to previous substrates because e.g., wooden strips were difficult to write upon and difficult to store to preserve records, as they had to be tied into bundles consuming much space. Nowadays, this flat configuration is still one of the main advantages for the design of electrochemical cells.

The process of paper manufacturing involves the mechanical or (bio)chemical conversion of a fibrous raw material (commonly wood) into pulp (free fibers separated to the unusable fraction) and later, bleaching and further treatment (with mineral fillers, polymeric additives…) depends on the type and grade of paper that is to be produced [44]. In the paper factory, the pulp is dried and pressed to produce paper sheets.

#### 2.1.1. Paper Source

**Figure 2:**
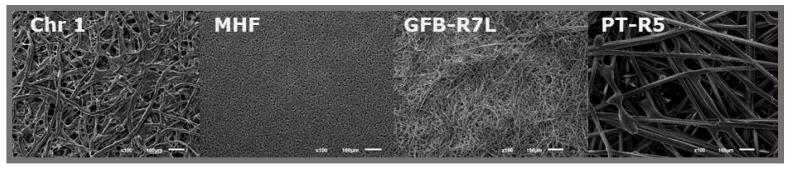
SEM images (zoom ×100) of different paper substrates: Chr 1: Whatman Grade 1; MHF: Millipore Hi-Flow nitrocellulose membrane; GFB-R7L: Mdi glass fiber; and PT-R5: Mdi polyester. Reprinted (adapted) from [40], Copyright (2017), with permission from Elsevier.

**Figure 3:**
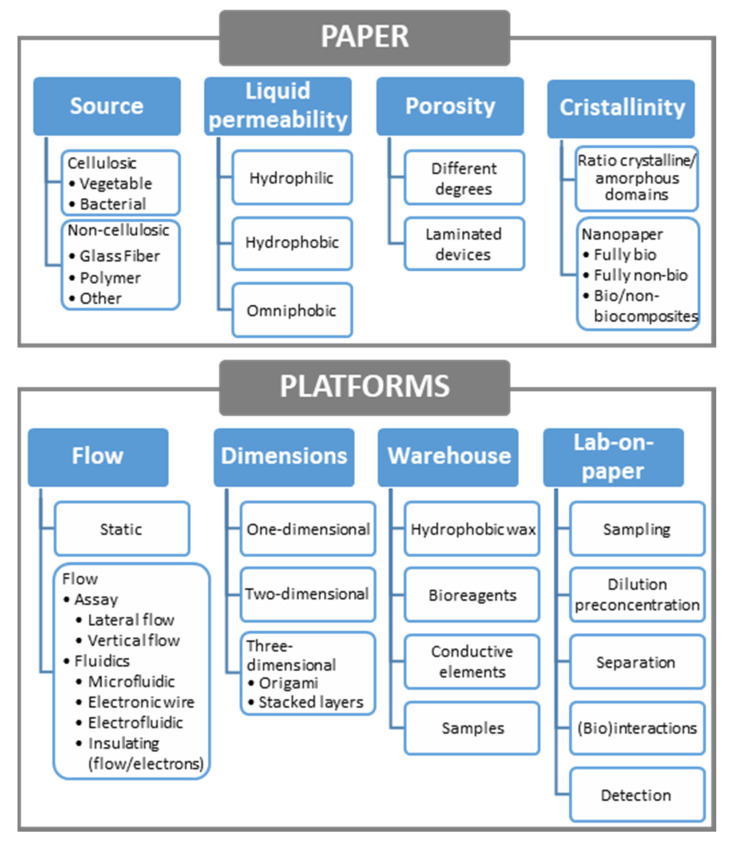
Classification of paper-based platforms according to different criteria and properties.

Although vegetable (and then cellulosic) materials are mainly considered when referring to paper, other materials such as e.g., glass or polymers that also form fibers (Figure 2) and can be pressed to form sheets, could also be included. Then, a first classification (Figure 3) would distinguish among cellulosic (based on cellulose, a polymer of ß-linked D-glucose units) and non-cellulosic paper, depending on its source. Both are very interesting, although cellulosic materials exceed, by far, the rest of paper materials. In each of the classes, subclasses could be made according to the different vegetable species (also bacteria) in the first or the different materials in the second one. Bacterial cellulose, an extracellular polymer produced by some microorganisms, chemically pure and with high water-holding capacity and mechanical stability, has been proposed to develop enzymatically active paper including lipase [45] and also lactate oxidase, in this case for electrochemical detection of lactate in sweat [46].

Cellulose can be also modified for changing the properties of the final product, being cellulose acetate or nitrocellulose the most common outputs. Cellulose acetate [47] is one of the most important esters of cellulose, obtained by reaction of cellulose with acetic anhydride and acetic acid in the presence of sulfuric acid. Nitrocellulose is the nitrate ester, obtained by nitrating cellulose with nitric and sulfuric acids. The analytical applications are numerous in both cases, as e.g., electrophoresis on cellulose acetate [48] or lateral flow immunoassays employing nitrocellulose membranes [49]. There are other different cellulose-based materials that can be employed for analytical purposes, such as the case of cellophane, brand name for cellulose films that are manufactured by regenerating cellulose from cellulose xanthate (viscose). It has been proposed for use in microfluidics either coated (with a micron thick coating of nitrocellulose or polyvinylidene chloride) or uncoated [50].

#### 2.1.2. Hydrophilicity

Cellulose fibers, the building blocks of paper, are hollow tubes (*ca.* 1.5 mm long, 20-µm wide, with a wall thickness of ca. 2 µm) that form a porous, hydrophilic material that take up more than their own mass of water [51]. However, although hydrophilicity is one of the main characteristics that defines paper, in some cases is not desirable. In the fabrication process, “sizing” agents can be employed to lower surface energy, increasing water-contact angle and lowering rates of water penetration [52]. Alternatively, cellophane or also pretreated cellulose-based paper, as in the case of this silanized with decyltrichlorosilane [53] to render it hydrophobic, or with fluoroalkyl trichlorosilanes to obtain omniphobic paper [54], could be employed. In this last case, the paper is both hydrophobic and oleophobic, repelling water and aqueous solutions containing ionic and non-ionic surfactants but also organic liquids or other complex liquids such as blood.

#### 2.1.3. Porosity

Surface chemistry and porosity influence wet properties of paper, very important especially in the development of devices for flow assays. In non-porous paper, only the macroscopic external surface is accessible. The availability of the inner surface and then, the specific surface area that is accessible, increases with porosity. Related to this, there are two correlated macroscopic properties of paper that have to be considered: thickness (in m) and basis weight (g.m^−2^), which is the mass of dry paper per square meter [51]. Using both parameters, the total pore volume can be estimated. In the case of the most commonly used paper in analytical devices (Whatman No. 1 filter paper, 180-µm thick with a basis weight of 87 g.m^−2^, and then a density of 483 kg.m^−3^), considering a density of the solid component of wood fiber of 1540 kg.m^−3^, the pore/volume ratio results approximately 0.69 [51]. This porosity arises from spaces between the fibers, uncollapsed fiber lumens, and the intrinsic porosity of the fiber walls.

Porosity changes depending upon tree species, pulping type and drying after pulping, which can cause some of the pores to collapse. Alternatively, the porosity of paper-based platforms can be mechanically tuned, as is the case of the laminated paper-based analytical devices [55], enclosed between plastic sheets using a roll laminator. Apart from providing mechanical strength to the paper device, compression changes the structure since lamination reduces the thickness of the paper and the effective pore size, and in turn, the flow rate. The Washburn’s equation [56] that describes the capillary flow in a bundle of parallel cylindrical tubes, can be extended to a paper strip: $L^{2}=\gamma Dt/4\eta$, where *L* is the length of the strip, γ the effective surface tension, *D* the pore diameter, *t* the time, and η the dynamic viscosity of the fluid, can be rearranged as L/t=γD/4ηL, where *L/t* is the flow rate. Its linear dependence upon the pore diameter (*D*) is clear. Although it could seem an inconvenience, decreasing the flow rate increases the residence time what could be interesting when using paper as a (bio)reactor. This useful equation indicates variables that alter linear velocity. Thus, to increase velocity, a high surface tension, low viscosity or low density (if the liquid runs upward) liquid could be employed [57]. On the other hand, the surface energy of paper can be lowered to increase the contact angle of a liquid, what can be achieved by depositing material into the pores or chemically bonding groups to hydroxyl groups on cellulose. As commented before, treatment with organosilanes in gas phase [54] is a way to increase the contact angle $\left(\theta_{H_{2}O}>>140^{{}^{\circ}}\right)$.

#### 2.1.4. Cristallinity

Another parameter that can be correlated to paper hydrophilicity is the degree of crystallinity of cellulosic fibers (usually 50%). Cellulose is made up of bundles of fibrils called microfibrils, each individual consisting of crystalline and amorphous regions. Crystalline domains do not swell with water but by contrast, amorphous cellulose swells in water and is more susceptible to chemical reactions. Depending on the applications, paper surfaces can be saturated with water that forms slightly anionic water swollen hydrogel of amorphous cellulose and hemicellulose. Then, negatively charged particles, polymers or DNA have little tendency to adsorb [51]. In this context, cellulose nanofibers and nanocrystals (rod-shaped crystalline part that remains after removal of the amorphous domains) can be extracted from vegetable cellulose to produce nanopaper with different properties [58]. Apart from the crystallinity, the size of cellulose microfibrils in a paper is in the micrometre scale and consequently their specific surface area and hydrogen bond intensity are much less than that of nano-sized cellulose fibres [59]. As happens with the definition of paper, nanopaper can also come from different sources. Being a thin sheet mainly made of tightly packed nanostructures, those based on cellulose, chitin or chitosan nanofibers, are considered as fully bionanopapers. At the other side of the classification are the fully non-bionanopapers, based on nanographene (oxide), silicon carbide, carbon nanotubes or even synthetic polymers (polyurethane, polypropylene, polyvinylfluoride, etc.). In between, mixed bio/non-bionanocomposites including a main matrix component with other secondary components embedded are possible. In all the cases, interesting applications are being developed, including also the incorporation of screen-printed electrodes [60].

The classification of paper-based platforms according to different criteria, including properties of the material, is summarized in the schematics of Figure 3.

### 2.2. Paper in (Electro)analytical Platforms

**Figure 4:**
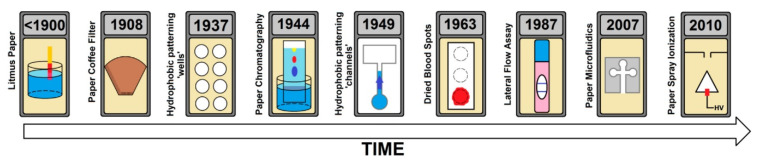
A history of paper in science, highlighting important milestones. Reprinted from [57] (open access, https://pubs.acs.org/doi/10.1021/acs.analchem.8b04825 accessed on 16 February 2021, further permissions should be directed to the ACS).

**Figure 5:**
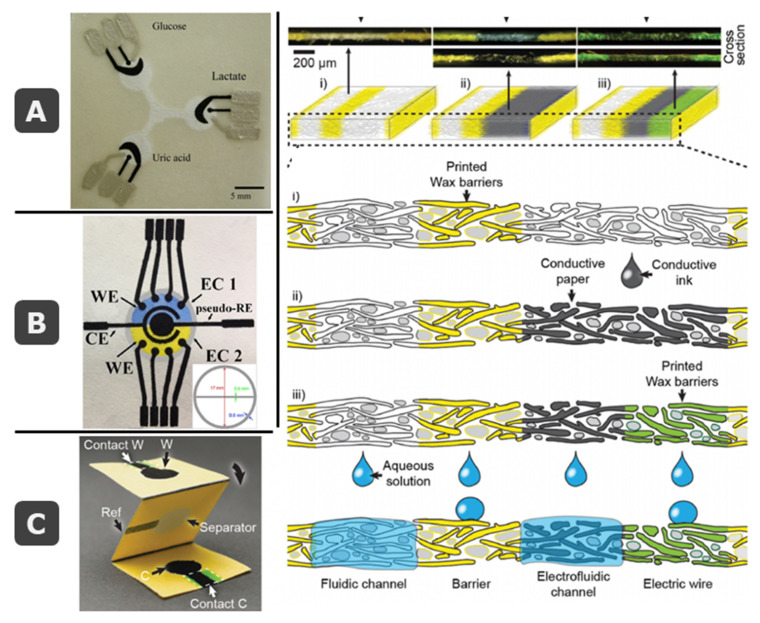
(**A**) Pioneer work on ePADs with screen-printed electrodes (SPEs) for multianalyte enzymatic detection of glucose, uric acid, and lactate. Reprinted with permission from [37]. Copyright (2009) American Chemical Society. (**B**) Picture of the paper-based electrochemical device developed by de Oliveira et al. with two electrochemical cells, with 4 working (WE) each and sharing reference (RE) and counter (CE). Reprinted from [65], Copyright (2019), with permission from Elsevier. (**C**) Integration of microfluidic and electronics, exemplified in an origami-based electroanalytical cell and visual explanation of the concept of electrofluidics. Reprinted with permission from [66], Copyright (2016) Wiley-VCH Verlag GmbH and Co.

The main use of paper in analytical (bio)platforms dates back to the 1930s with the development of paper chromatography [61], although estimation of pH with litmus paper and employ of paper as filter are even older (see Figure 4, [57]). Home-based pregnancy tests were based on the use of porous materials and pioneered analysis decentralization in the 1980s [62]. An impressive renaissance was done by Whitesides’ group who patterned microfluidic channels in two dimensional platforms for multianalyte clinical analysis [32]. They were devised in a time when the need for cheap diagnostics in low-resource settings was urged [63]. The developing world does not have access to many of the best medical diagnostic technologies; these require air-conditioned laboratories, refrigerated storage of chemicals, a constant supply of calibrators and reagents, stable electrical power, highly trained personnel, and rapid transportation of samples. In remote zones, running water and electricity may or not be available, power is at best intermittent with wide fluctuations in voltage. The ambient temperature can range from 10 °C to more than 40 °C. Dust, wind and contaminating pathogens are very common. Potentially high-risk human samples are routinely handled with few precautions. Maintaining and calibrating even moderately complex instruments still presents a challenge. Actually, microfluidic paper-based analytical devices (μPADs) were conceived for useful application to the diagnostics in the developing world [33]. The scheme in Figure 4 shows the milestones in the history of paper science, with special focus on analytical devices. It includes the paper spray ionization, where the sample is deposited onto a sharp tip cut out of paper, aligned in front of a mass spectrometer [64] and should be completed with the introduction of the electrochemical detection by Henry’s group in 2009 [37] to generate electrochemical paper-based analytical devices (ePADs) (Figure 5A). This detection, initially based on the use of screen-printed electrodes, the topic of this revision, fits perfectly with the concept of decentralization analysis.

#### 2.2.1. Configuration: Static or Flow Assays

Hydrophilicity and porosity, commented in Section 2.1, are the main causes of capillary forces, in such a way that a liquid can easily flow through the gaps in porous media without using any extra energy due to the inherent capillary action. Then, paper allows passive transport of solutions and also can be used to absorb liquids. Then, both static and flow analytical devices are possible, as shown in the classification of Figure 3. In the first case, a volume of liquid can be deposited over a delimited hydrophilic area [42] or aspirated through a sampler [67] to perform the analysis. In the case of flow assays, lateral or vertical flow platforms [68] have been designed. Lateral flow platforms have been the pillar of rapid point-of-care diagnostics due to its simplicity, rapid process, and low cost. However, vertical (or flow-through) assays have emerged as an interesting alternative due to its rapidity and design possibilities.

#### 2.2.2. Dimensions

Additionally, a basic classification of analytical platforms can be made according to their dimensions. One-dimensional channels can be made from paper sheets in two ways: by cutting two-dimensional paper to obtain physical boundaries or by forming hydrophobic boundaries through impregnation of agents. In both cases the methodology can be simple and cheap or more complicate and expensive. Precision depends also on the procedure (handmade or automatized). Thus, scissors and razor blades could be used as well as craft-cutting machines or lasers for delimiting the paper-based platform. In this case, due to the fibrous structure, paper tearing can occur and due to the fiber orientation, different profiles are obtained when cutting is made in parallel to the *y*- or *x*-axis, which have to be previously considered since fluid transport may depend upon the angle at which the paper strip is cut. Additionally, the *z*-axis (thickness) has to be taken into account, since the mass distribution in a paper sheet is usually not constant, with the maximum density in the center and decreasing near the surfaces [51]. One of the best examples of one-dimensional platforms are those employed for already introduced lateral flow immunoassays [69], where after assembling all the components required for the assay on a two-dimensional adhesive backing (i.e., sample pad, conjugate pad with detection antibodies, nitrocellulose membrane with test and control lines of immobilized capture antibodies, and absorbent pad) different units are cut. Being one-dimensional is one of the main advantages of other low-cost platforms, e.g., thread-based devices, allowing not to delimit channels in colorimetric [70] or electrochemical, either amperometric [71] or potentiometric [72] platforms. However, when delimiting a hydrophilic working area (by photolithography, wax printing, etc.) is required in paper-based devices, a two-dimensional structure usually is employed, including in most of the cases several one-dimensional channels. Apart from this, the flat structure of paper allows easy generation of 3D platforms, by stacking layers of similar or different composition (e.g., adhesive tape, polymeric films, etc.). Separate layers can be piled up or, alternatively, taking advantage of the foldability of paper, origami-based devices [50,73] can be employed, as well as other “pop-up” electrochemical platforms [74]. In this last case, a single sheet of paper is folded into a three-dimensional device that changes the shape and integrates fluidic and electrical connectivity by simply folding and unfolding the structure. The reconfigurable 3D structure provides mechanical support and allows control timing. Similarly, a sliding strip microfluidic device enables perform colorimetric ELISAs on paper [75]. The possibilities of multilayer devices are enormous. An interesting example is the use of both paper and hollow channels in the same analytical platform [76,77]. Removing the cellulose matrix from within a predefined channel and leaving the bottom of the channel hydrophilic, the flow rate of solution in the channel can be enhanced without requiring external equipment. In this way, analysis time is reduced, and larger fluidic networks can be employed. Non-specific adsorption is decreased, and micrometer-sized objects could flow freely. Colorimetric [77] and electrochemical [76] detection have been demonstrated, in the last case for voltammetry and amperometry, under flow and no flow conditions.

#### 2.2.3. Functions: Lab-on-Paper Devices

These multilayer devices (made of a folded continuous layer or several piled up) allow the integration of different functions, approaching the concept of lab-on-paper. Although these could be included in single-layer devices, the use of several layers brings more possibilities. The analytical process is that performed with the aim of obtaining information that allows solving social, economic, scientific, or technical problems. The analysis can be qualitative (with a binary yes/no answer obtained) or (semi)quantitative. Steps go from sampling to data treatment, including detection and several operations carried out with the aim of obtaining sensitive and selective methodologies (here most separation steps are included as well as (bio)interactions for recognition purposes or to obtain detectable species). Technological improvements, especially in sample processing, fluid flow control, signal amplification and component integration increased the applicability of PADs to real-world problems [78]. Several strategies can be employed for controlled fluid actuation and manipulation and thus, slipping, channeling, delaying delivery of reagents, switching to initiate flow on demand, or fluid mixing (considering that the flow is generally laminar) is possible. Moreover, inclusion of nano- and microsized objects as well as filtration [79] or separation [80], and also dilution or preconcentration [81], have been demonstrated.

One of the reasons why paper is excellent substrate for the development of analytical platforms is its versatility. Many different systems and applications can be converted into paper-based formats, taking advantage of its tunable properties. As an example, regarding the detection, multiplexed devices including several electrochemical cells with multiple electrodes can be easily designed [65] (Figure 1B).

#### 2.2.4. Paper as a Warehouse

In a more general way, paper can be considered as a huge warehouse where different elements can be stored using weak or strong bindings, mainly: (i) hydrophobic materials, already commented, to delimit microfluidic areas in two-dimensional platforms, (ii) biological materials, as active reagents for the development of bioassays, (iii) samples that could be even dried for further analysis, allowing transportation from remote settings [82], and (iv) conductive materials, very interesting for electroanalytical purposes. Combination of some of them introduces the concept of electrofluidics [66], by monolithic integration of microfluidics and electronics (Figure 5C). Thus, in the same paper device a hydrophobic barrier (insulated non-microfluidic electrical conductor), and microfluidic channels with and without electronic conductivity could coexist by modification with wax and carbon nanotubes (or conductive polymers). In this case, since an aqueous dispersion of conductive elements is employed, the flow is not impeded, what could happen when dense inks are employed. The same idea could be exploited using micropatterned conductive structures where the conductors (carbon nanotubes or silver nanowires) are monolithically integrated with nanocellulose-based paper [83].

Introducing electrode integration and coming back to the history of papermaking, even when paper dates back from year 105, printing was long retarded, with the first text printed upon paper finally completed in the year 770 by Empress Shotoku of Japan, more than 665 years later [43]. The use of a soft and pliable paper made possible to make an impression from a wood block spread with pigment. After putting the paper over the block, a ball was rubbed by hand until a definite impression was made upon the paper. In Europe, fibers from macerated linen and cotton cloth formed sheets that were impregnated with gelatin when Gutenberg established his printing office in Mainz using metal blocks. Similar to what happens nowadays, the type of paper influenced the process and readout. Thin and transparent Oriental paper allowed writing on one side, meanwhile thick and opaque European paper allowed writing or printing on both sides. Obviously, the process has evolved and improved with time and nowadays, screen-printed thick-film technology is one of the more successful in the development of electroanalytical devices, also in paper-based devices, as commented in more detail in the following section. Actually, the first electrochemical cell for paper devices was made by a screen-printing process of conductive inks [37]. Since fibers are pressed and acquire a flat configuration, paper is very appropriate for integration of electrochemical cells, in a similar way that is done for the rest of the substrates. However, similarly to what happens at the beginning of the process, as paper is a porous substrate, if an electrolyte is employed, ionic conduction exists between the two faces. Then, electrodes do not need to “share” the same surface, and screen-printed technology, that commonly uses a pattern to locate all the electrodes required for electrochemical readout in the same face, is not required. Simple deposition of ink in a wax-delimited area, without the need of screens or stencils, can be employed. Two more pins are used as electrodes that connect the electrolyte by the other side (Figure 1D, [42,67]). However, since this technology is commonly employed with non-porous substrates (as ceramics or polymers), the translation into paper was done performing the same procedure, considering paper as one more flat surface with unconnected faces. However, this is a very differential advantage when compared to the rest of substrates employed in SPEs. Moreover, printing and coating technologies are based on the application of almost any fluid onto dry paper. Aqueous or organic solutions could be employed. Usually, aqueous solutions are particularly easy because capillary forces and the hydrophilic nature of cellulose promote rapid sorption, but in SPE technology conductive elements are dispersed in organic solvents and curing is necessary. Moreover, biomolecules can be also spotted or printed onto dry paper without denaturation.

## 3. Paper-Based Screen-Printed Electrodes

As mentioned before, besides being a flat substrate, paper can have other utilities such as storage of reagents or sample [84,85], support for (biological) reactions [42,86,87,88,89], or platform for taking [67,90] or treating the sample (e.g., preconcentration [81,91] or separation [79,92,93]). Taking this into account, paper-based electroanalytical devices integrating SPEs can be designed in different formats: (i) combining paper with a SPE card fabricated on ceramic or polymeric materials [79,84,85,86,87,88,89,91,92,94,95,96,97,98,99,100,101,102]; (ii) combining one electrode of the electrochemical cell (e.g., WE) made on paper with other electrodes (e.g., RE and CE) of a SPE card printed on a conventional material [42,103,104,105]; and (iii) printing the SPE directly on paper [5,106,107,108]. In the last case, 2D devices are the most basic but, by stacking and/or folding the paper along the vertical axis, devices with 3D formats (multilayer and origami) can be easily constructed. Obviously, the fabrication of 2D devices is much simpler but 3D platforms can improve analytical characteristics (mainly reproducibility and sensitivity) and reduce the steps of the analytical procedure as well as time analysis [108]. Here, electrodes could be included in the same or different layers.

### 3.1. SPE Cards Combined with Paper Elements

In the case of the lowest degree of integration (paper and SPE ceramic card), the paper is mainly used as support for biological components (enzymes [85,86,95,97,98,99], but also antibodies [87] or nucleic acids [88]) where reactions with components of the sample take place. Paper was also used as a medium for cell culture. In this case, the SPE platform allowed the on-line evaluation of cell viability by monitoring dopamine release from cell damage models [109]. Alternatively, the paper was also combined with a SPE card for sampling, as in the tear sampling system developed by Honikel et al. using Whatman No 41 ashless filter paper [90].

**Figure 6:**
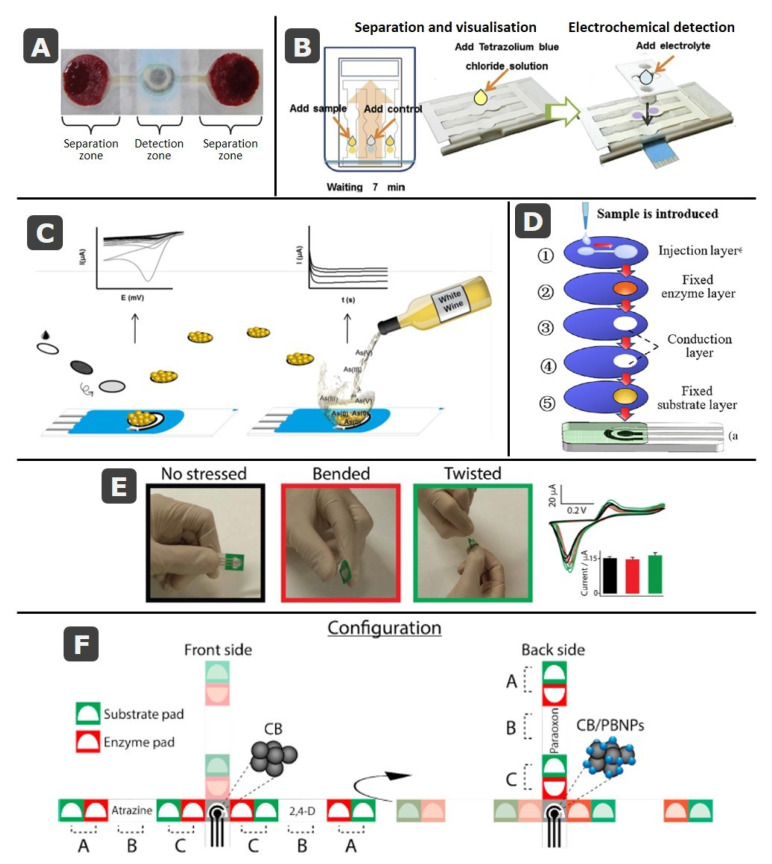
SPE cards combined with paper elements: (**A**) Picture of the paper-based device developed by Noiphung et al. for whole blood separation and glucose determination. Reprinted from [79], Copyright (2013), with permission from Elsevier. (**B**) Schematic representation of the separation, visualization and measurement steps in the paper-based devices developed by Primpray et al. for the determination of steroids. Reprinted from [92], Copyright (2019), with permission from Elsevier. (**C**) Schematic representation of the paper-based system for in-situ generation of gold nanoparticles and further analysis of arsenic developed by Nuñez-Bajo et al. Reprinted with permission from [105]. Copyright (2017) American Chemical Society. (**D**) Schematic diagram of the multilayer platform developed by Yang et al. for pesticide detection. Reprinted with permission from [95], Copyright (2020) Wiley Periodicals LLC. Fully-integrated paper-based electrochemical devices: (**E**) Evaluation of the robustness of the paper-based device developed by Cinti et al. for the detection of chloride ions, after 100 repeated bending (red) and twisting (green) tests. Reprinted from [114], Copyright (2017), with permission from Elsevier. (**F**) Schematic diagrams of the configuration of the origami device developed by Arduini et al. for the detection of pesticides. Reprinted from [115], copyright (2018), with permission from Elsevier.

In a following step of the analytical process, the assembling of layers of different types of paper has been also used for the treatment of the sample. A clear example of this application is the analysis of whole blood as in the glucose sensor developed by Noiphung et al. [79] in which Whatman No. 1 and blood separation paper, VF1 and VF2, were combined with a Prussian Blue screen-printed electrode. A wax-dipping technique was used to design microfluidic patterns that consisted of two separation zones and one more for detection, where the enzyme glucose oxidase was immobilized (Figure 6A). Paper can be employed for separating analytes before their detection as in the microfluidic platform developed by Primpray et al. [92]. This device used Whatman SG81 paper, which is an ion exchange paper that combines cellulose with large pore silica gel, for the separation of dexamethasone or prednisolone steroids (Figure 6B). Moreover, and taking advantage of the white color of paper and of the intense blue color produced by the interaction of tetrazolium blue with steroids, this reagent was added to the channels with the aim of visualizing the exact position of each steroid, facilitating the subsequent electrochemical detection [92]. Lateral flow immunodevices are also composed of different layers overlapping one another. Then, screen-printed electrodes can be included just below the test line (this containing the antibody to capture the analyte) of the membrane for detection, in this case taking advantage of the vertical flow [110], or even on the same membrane [111,112]. Alternatively, another possibility is to include the capture antibody directly over the screen-printed working electrode in a mixed biosensing approach [113]. Apart from these examples in which a lateral flow is produced, the combination of paper-based working electrodes with electrodes from screen-printed cards have been used in static systems with the aim of reducing the cost by reusing the RE and CE of the same SPE for different paper WEs [42,103], but also for the in situ electrogeneration of nanoparticles on paper-based carbon [104] or gold [105] electrodes, the last one employed for the chronoamperometric determination of arsenic in white wines (Figure 6C).

On the other hand, several layers of paper can be added in vertical flow configurations, with each layer for a different function as in this multiplex platform developed by Yang et al. for the detection of organophosphorus pesticides [95]. The detection and identification of the pesticides were performed recording impedance time-sequence spectra after inhibition of the enzymatic activity of acetylcholinesterase by pesticides. This platform consisted of five layers for: (1) injection, (2) enzyme immobilization, (3 and 4) sample transport, and (5) substrate (indophenol acetate) immobilization (Figure 6D). The layers 3 and 4 were hollow and their function was to allow interaction between sample and enzyme for a long enough time. In a similar way, stacking different layers over a SPE has been employed for electrochemical detection of specific sequences of DNA by combining recombinase polymerase amplification with an electroactive mediator [116]. In this case, a disposable paper strip that incorporates a carbon SPE card is included in a handheld device that accomplishes thermoregulation and enables electrochemical detection of *Mycobacterium smegmatis* and *M. tuberculosis*. On the other hand, origami paper devices (folded multilayer platforms) can be also combined with SPEs printed on ceramics or polymers [85,117] as in the case of the device developed by Pinyorospathum et al. [117] for the determination of human C-reactive protein (CRP), which is an important biomarker for different cardiovascular diseases. This platform consisted of a SPE printed on a PVC substrate, that was wrapped in between the two paper folded layers containing reagents for detection.

### 3.2. Fully-Integrated Devices: Electrochemical Cells Printed on Paper

Although there are numerous different designs that combine SPEs (in ceramic/polymer substrates) with paper devices, the most integrated approach is achieved by printing electrodes directly onto the paper creating 2D and 3D devices, with all the three electrodes in the same or different layers [78,108]. In two-dimensional devices, the sample is deposited in the same layer in which the electrochemical cell is printed or flows toward it by capillarity. As commented before, platforms are commonly fabricated by patterning a single piece of paper with a hydrophobic material (e.g., wax or photoresist) that delimits hydrophilic microfluidic channels, areas or reservoirs [118,119,120,121,122,123,124,125,126,127,128,129,130,131,132,133,134,135,136]. Other materials such as adhesive tape can be used either as an additional stencil for printing the electrodes [121,137] or to delimit the area of the working electrode once the ink has been spread on a specific piece of paper [138]. In this last case, screen or stencil printing is not necessary since the ink is directly deposited on a piece of paper. Delimiting the area of the working electrode with hydrophobic wax would avoid the need of an additional tape layer [42,67]. Different specific (bio)reagents (enzymes [129,130], antibodies [139], nucleic acids [120,131,140]), or nanomaterials [124,134,141]), which react with the sample, can be immobilized on delimited areas. On the other hand, multiplexed devices can be easily designed [65]. The combination of paper and screen-printed technology allows to obtain flexible devices, able to be bended or even twisted without loss of analytical signal as in the case of this designed by Cinti et al. [114] (Figure 6E).

Taking advantage of how easy it is to fold, stack, or cut the paper, there was a subsequent development of 3D multilayer and origami paper-based devices [73,93,117,142,143,144,145,146,147,148,149,150,151,152,153,154,155,156], including integration of electrodes directly on paper. These seem to require more laborious procedures of construction but the use of software for transferring wax and ink designs to paper makes this an easy task, especially useful when more sophisticated operations are enabled: e.g., sampling [143,157,158], sample delivery [146,159], or sample treatment [93]. As mentioned before, in many of these devices, the electrodes of the electrochemical cell are printed on different layers of the devices with the aim of improving the contact of the electrodes with the sample or decreasing the size of the device [73,142,144,152,160,161,162,163,164,165,166]. Moreover, vertical microfluidics, in comparison with lateral flow, helps to reduce the consumption or reagents and the loss of sensitivity due to the diffusion of the analyte. An example of these origami devices is the one developed by Arduini et al. for the multiplexed detection of three pesticides (paraoxon, 2,4-dichlorophenoxyacetic acid and atrazine) of different classes exploiting their capability to inhibit the activity of enzymes butyrylcholinesterase, alkaline phosphatase and tyrosinase [115]. The configuration of this device can be seen in Figure 6F. It consisted of two office paper-based SPEs cells, printed on the front and the backside of the device. Different filter paper strips were combined with these electrodes using adhesive tape, with the aim of determining the initial and residual enzymatic activity and therefore, the concentration of the pesticides. Each filter paper strip (from a total of three) was constituted of two pairs of different pads (A and C in the figure), one for containing the enzyme, and the other the substrate, separated by an empty paper. To determine the initial enzymatic activity, enzyme- and substrate-pads were contacted, and distilled water was added to perform the measurement. After cutting the first used pads, the sample was analyzed putting in contact the other enzyme- and substrate pads of the following strip and letting the sample incubate. After addition of water, the analytical signal was recorded. This device performs 6 measurements for recording the signals corresponding to the three pesticides [115].

Origami devices have been widely developed for clinical and biological applications [5,106,107]. For many of them, biological reagents such as enzymes or antibodies are used but those based on molecularly imprinted polymers (MIPs) are also useful approaches [121,137,155]. Amatatongchai et al. developed a MIP-based origami paper using alkyl ketene dymer (AKD)-inkjet printing to create circular hydrophobic areas [155]. The graphite working electrode of the screen-printed electrochemical cell was modified with a Fe_3_O_4_@Au@SiO_2_-MIP nanocomposite, showing electrocatalytic activity toward the oxidation of serotonin, determined by linear sweep voltammetry in pharmaceutical capsules and urine samples [155].

**Figure 7:**
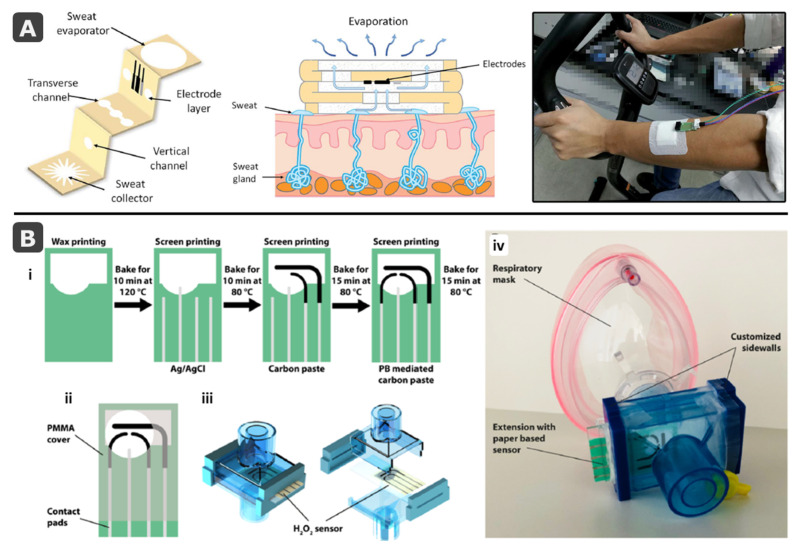
(**A**) Schematic diagram of the 3D microfluidic device developed by Cao et al. for sweat analysis. Schematic representation of the device applied on skin; a channel was formed by folding the origami device through which the sweat flow from the skin to the electrochemical cell. Picture of the device applied on the forearm of a user. Reproduced from [158] with permission from The Royal Society of Chemistry. (**B**) Schematics of the procedure of fabrication and the final design of the screen-printed electrochemical cell inserted in the mask developed by Maier et al. (**i** and **ii**). Model of the customized filter extension in which the screen-printed electrochemical cell is inserted (**iii**). Photograph of the respiratory mask with the filter extension containing the screen-printed electrode (**iv**) Reproduced from [167] (open access).

Foldability is also highly useful for the creation of wearable devices for on-site monitoring of important parameters in non-invasive samples. One example is the origami paper-based platform developed by Cao et al. for the determination of glucose in sweat [158]. This 3D-microfluidic device was fabricated patterning the paper with wax and folding it to form five layers with different functions: sweat collector, vertical and transverse channels, electrode layer, and sweat evaporator (Figure 7A). In this way, sweat was absorbed by the collector and flowed toward the electrochemical cell by capillary forces. Sweat evaporation on the evaporator allowed continuous flow keeping fresh sample flowing across the electrochemical cell and avoiding sweat accumulation [158]. An on-body test was carried out to validate the device, but still the stability has to be evaluated and the size reduced. On the other hand, using 3D or origami approaches, paper-based SPEs can be directly integrated in wearable devices as in the case of the mask developed by Maier et al. for the continuous real-time monitoring of exhaled hydrogen peroxide (H_2_O_2_), an important biomarker in respiratory diseases [167]. The electrochemical cell was printed on a wax-patterned paper in which a solution of electrolyte was evaporated to form a solid electrolyte. Two carbon working electrodes were printed: one with Prussian Blue as mediator, for the H_2_O_2_ detection, and the other without modification, for subtracting the background signals (Figure 7B). This screen-printed electrochemical cell was placed inside a respiratory mask so the user breath directly onto it. This sensing mask was tested in simulated breath.

In a similar way, the high versatility of screen-printing technology, which allows to produce miniaturized electrodes, is also applicable not only to paper and other two-dimensional materials such as transparency sheets [31,168,169,170], but also other such as textiles or plastics, leading to a wide variety of wearable devices [106,171,172,173] such as bandages [174,175,176], gloves [177], rings [178,179], or pacifiers [180].

## 4. Conclusions and Perspectives

Paper has demonstrated to be an excellent substrate for the design of extremely innovative analytical platforms. The thick-film technology of screen-printing fits perfectly with this material that, in turn, is very appropriate for electroanalysis. Paper is an excellent container for both electrodes and electrolytes required for interfacial techniques. The possibilities are enormous, and the classification includes many varieties according to paper properties (porosity, crystallinity, hydrophilicity, etc.) and platform characteristics (dimensions, flow, etc.). Most of them have been applied to the clinical field and a challenge is to expand their utilization to other areas. Actually, paper-based electroanalytical devices would be very useful to the agri-food sector, to evaluate the presence of certain molecules and/or the freshness of a beverage/foodstuff [6,7,8]. In this way, these devices could reduce the gap between complex laboratory analysis and simple point-of-need testing, lowering costs, simplifying procedures, and reducing waste generation.

As reported in the bibliography as recommendation for future paper-based research by Verpoorte’s group [57], if these devices are to provide society with the tools to perform on-site analyses, one have to learn from previous successes (e.g., regarding LFAs, avoiding the use of pipets for sample application (as e.g., in [67]) or of complicated readout systems) and look beyond the piece of paper to increase the functionality, by including various elements or by integrating electronics (as e.g., in [66]). Paper is so versatile that true lab-on-paper platforms are possible by incorporating the different steps of the analytical process. Moreover, combination with portable electrochemical readers approaches the analysis to the point where is required. It is possible to find nowadays potentiostats in the “do-it-yourself” format [181,182,183], with wireless control from smartphones [184,185], or wired in case of older mobile generations [186]. Apart from the instrumentation, other components required such as the energy source could also be incorporated in paper format, as e.g., paper-based fuel cells [187]. Paper potentiostats have also been suggested for integrated biosensing [188], even including a screen-printed manganese dioxide battery and an electrochromic display, which approaches the concept of “use-and-throw” instruments in what can be considered as promising paradigm-changing new products [189].

Notwithstanding the above, the commercialization of these devices continues to be a challenge since the knowledge transfer from laboratories to the society is still complicated. Although general printed electrochemical devices represent a unique opportunity to enable low-cost, fast, non-invasive and/or continuous monitoring of analytes, metrological aspects such as sensitivity, repeatability and stability represent very challenging aspects [190]. It is important to note that, in the laboratory, many of the fabrication and assembling steps are handmade, which affects the precision. Therefore, automatization and mass production could help to improve it. Although operational stability is not a concern when single-use platforms are aimed, an important issue is storage stability, especially relevant when biological reagents are involved. However, despite these yet unmet challenges, the potential of paper-based printed devices is clear as well as the relevance they will have in the future [191]. In an increasingly knowledgeable society with growing globalized problems, as is the current pandemic, simple tools that provide fast, reliable, and on-site responses will be undoubtedly, and increasingly required.

## Data Availability

Not applicable.
